# College and Hospital Notes

**Published:** 1909-09

**Authors:** 


### COLLEGE AND HOSPITAL NOTE.

LORD STRATHCOMA has given $500,000 to McGill University. Of this amount, $450,000 is to be used in completing the new medical building and $50,000 in augmenting professors' salaries.

